# Backchannel Behavior Influences the Perceived Personality of Human and Artificial Communication Partners

**DOI:** 10.3389/frai.2022.835298

**Published:** 2022-03-30

**Authors:** Peter Blomsma, Gabriel Skantze, Marc Swerts

**Affiliations:** ^1^Department of Communication and Cognition, Tilburg School of Humanities and Digital Sciences, Tilburg University, Tilburg, Netherlands; ^2^Department of Speech, Music and Hearing (TMH), KTH Royal Institute of Technology, Stockholm, Sweden

**Keywords:** embodied conversational agent (ECA), backchannel behavior, personality and behavior, conversational AI, o-cam paradigm

## Abstract

Different applications or contexts may require different settings for a conversational AI system, as it is clear that e.g., a child-oriented system would need a different interaction style than a warning system used in emergency situations. The current article focuses on the extent to which a system's usability may benefit from variation in the personality it displays. To this end, we investigate whether variation in personality is signaled by differences in specific audiovisual feedback behavior, with a specific focus on embodied conversational agents. This article reports about two rating experiments in which participants judged the personalities (i) of human beings and (ii) of embodied conversational agents, where we were specifically interested in the role of variability in audiovisual cues. Our results show that personality perceptions of both humans and artificial communication partners are indeed influenced by the type of feedback behavior used. This knowledge could inform developers of conversational AI on how to also include personality in their feedback behavior generation algorithms, which could enhance the perceived personality and in turn generate a stronger sense of presence for the human interlocutor.

## 1. Introduction

### 1.1. Personality Perception

Personality refers to the consistent behavioral responses of a person and is often expressed in terms of the Big Five theory (John et al., 2008). There is a growing scientific interest in rendering conversational AI systems with various types of personality as this may help to make interactions with such systems more natural, and would allow to tune their interaction style to different situations or users. Our current paper will tackle this issue in view of the further development of so-called Embodied Conversational Agents (ECAs), i.e., computer interfaces that are graphically represented as a human body or human face, in order to allow users to interact face-to-face with computers in a way that resembles that of their interactions with real humans (Cassell, 2001; McTear, 2020).

Technically speaking, ECAs are nothing more than a collection of algorithms that together orchestrate the interaction with the interlocutor. To create the illusion that the interlocutor is not conversing with just some mindless algorithms, but with a partner who has thoughts and emotions, there have been attempts to render such conversational AI systems with a specific personality. This may enhance a feeling of social presence, and therefore increase the experience of dealing with a system that truly understands the intentions and feelings of the user (Biocca, 1999; Lee and Nass, 2003; Natale, 2020). Furthermore, it may be useful if conversational AI systems adapt their personality and conversational style to the specific application or intended audience. For instance, a conversational AI implemented for a playful environment would typically demand a different interaction style than one which is put to use in a crisis or emergency context (Goetz et al., 2003). Likewise, a conversational AI may have to adjust its behavior depending on whether it addresses a child or an adult, or a person with specific communicative deficiencies (Williams et al., 2021). Adaptation of its personality to the personality of the interlocutor could also increase conversational quality by exploiting the mechanics behind similarity-attraction, as indeed people have been shown that people feel more attraction toward people or systems that match their personality (Lee and Nass, 2003). Therefore, such adaptation could lead to more engaging conversations (Tapus and Matarić, 2008) and positive perceptions of the system (Andrist et al., 2015).

While personality potentially may help facilitating social presence and conversational quality, it would also seem to be a requirement to create next-level conversational AI systems in yet another sense. One of the factors that prevents the creation of human-like systems whose appearance is perceived as being similar to that of real humans is related to the uncanny valley effect, the phenomenon that small errors in behavior generation of the system can evoke feelings of fright discomfort in the interlocutor. While various theories exist regarding the cause of this effect, some explain it by cognitive dissonance (Yamada et al., 2013), i.e., the discomfort that arises because it is unclear to a user or observer if the conversational system should be perceived as human- or system-like. A conversational AI system that lacks a personality, and may therefore generate inconsistent behavior, could increase the feelings of unease on the part of the user, as he or she may feel uncertain on how to deal with a conversation partner who displays deviant interactive behavior (Zibrek et al., 2018).

### 1.2. Variability in Feedback Behavior

The current paper focuses on variability in feedback. In particular, we look at backchannel behavior, which refers to the feedback dialogue partners give each other on the smoothness of the information exchange process (Clark, 1996). While one person is talking, the addressee typically returns brief responses, called backchannels, which can be auditory (e.g., “uhuh”) or visual (e.g., head nod) in nature (Duncan Jr, 1974). Backchannels serve as cues to signal how the information was received at the other end of the communication channel, where one could roughly make a distinction between “go-on” or “do-not-go-on” signals. Although feedback behavior is person-dependent, backchannels are more expected at certain points in the conversation, namely during backchannel opportunity points (BOPs) (Gratch et al., 2006), moments when its appropriate to give feedback (Yngve, 1970). BOPs are signaled by the speaker with a so-called backchannel-inviting cue (Gravano and Hirschberg, 2011), signals via the prosodic channel, such as rising and falling intonations (Gravano and Hirschberg, 2009), low pitch ranges (Ward, 1996) and short pauses (Cathcart et al., 2003). However, addressees may vary regarding the extent to which they react to such speaker-initiated cues and utilize BOPs.

Behavior during those BOPs differs significantly from behavior outside of the BOPS. Specifically, speakers' nodding behavior, vocalizations and the use of the upper lip raiser (AU 10) is more apparent during BOPs than outside of those moments. During BOPs, people diverge considerably in timing, frequency and type of audiovisual feedback behavior. There are both differences between how people react to the same BOP (within-people diversity) and how the same person react to different BOPs (within-person diversity) (Blomsma et al., submitted). Also people can diverge considerably in timing, frequency and type of audiovisual feedback behavior. It is intuitively clear that, likewise, different conversational AI systems may have to vary regarding the degree, the type and the frequency of backchanneling. For instance, an “emphatic” tutoring system that has to assist learners to acquire a specific new skill may have to produce more supporting cues than a more neutral system that is consulted to give legal advice or specific route directions.

The current article therefore focuses on whether personality perception is influenced by the aforementioned variability in feedback behavior a person gives. This question is inspired by the outcome of our previous study, which led to the impression, though not explicitly tested in that earlier study, that differences in interaction style generated variable perceptions of the personality of the people whose feedback behavior was being recorded. While some participants appeared to come over as introvert and somewhat uninterested, others gave the impression of being extravert and lively, suggesting that there may exist a relation between the type of feedback behavior and the perceived personality of a person.

There are reasons to believe that this variability in behavior would lead to different personality perceptions. Although existing research into this question is scant, there are some studies that point into an affirmative direction. For example, Huang and Gratch (2012) analyzed the personalities of backchannel coders and the relation between that personality and the number of identified backchannel opportunity points. They found that a high number of identified backchannel opportunity points are related to high values for Agreeableness, Conscientiousness and Openness. Jain et al. (2021) showed that extraverted people have a higher tendency to use multimodal backchannels (e.g., a combination of utterance and nod), compared to introverted people who tend to rely on unimodal backchannels and show as well that this difference was perceived by human interlocutors when this behavior was re-enacted by a digital human-like robot. Bevacqua et al. (2012) found that extraverted persons produce more backchannels than introverted persons.

Albeit that feedback behavior is person-dependent and related to personality, we still lack knowledge on the perceptual impact of those person-dependent behaviors (e.g., what the perceived difference is between a passive vs. a dynamic addressee). If indeed the perception of personality is related to the type of feedback behavior a person produces, then this would give developers another opportunity to perpetuate the personality of a conversational AI system. Moreover, if feedback behavior is chosen randomly, such behaviors might conflict with the desired perceived personality of such conversational AI system.

### 1.3. Current Study

In this study, we thus would like to gain insight into the relation between perceived personality and feedback behavior. We will utilize participant recordings from a previously conducted o-cam based experiment (Goodacre and Zadro, 2010; Brugel, 2014), where each participant was made to believe to have a real-life conversation with another person, but who in reality is a pre-recorded speaker. In our first experiment, parts of these participant recordings are shown to observers who are asked to rate perceived character traits of the participant in the recording. Those ratings are analyzed in relation to the listening behavior of those participants (i.e., the auditory and visual backchannels during the recordings), to establish whether perceived character traits correlate with patterns in audiovisual backchannel behavior. In a second experiment, the same stimuli are re-enacted by a conversational AI (i.e., on a virtual Furhat robot), to verify whether the assessment of the personality of these synthetic characters is likewise affected by their feedback behavior.

## 2. Materials

For our study we utilized the dataset that was (partly) generated in a previous study described in Blomsma et al. (submitted). The materials consisted of video recordings of 14 participants (henceforth called original addressees) of an o-cam experiment and the identified backchannel opportunity points (BOPs) of those recordings. BOPs are moments during the conversation that allow for listener feedback from the original addressee (Gratch et al., 2006). For every BOP, the following behavior was encoded: (1) vocalizations: did an original addressee vocalize during the BOP or not, (2) the nodding behavior of the original addressee, quantified by amplitude (the maximum head movement angle in head-pose elevation direction) and frequency (number of upward and downward peaks per timeframe) and (3) the average contraction of a facial muscle called the upper lip raiser, as defined by the Facial Action Coding System as Action Unit (AU) 10 (Ekman et al., 2002). Blomsma et al. (submitted) found that those four variables (amplitude, frequency, vocalizations and AU10) were significantly different for original addressees during BOPs, as compared to the rest of the interaction.

The videos were recorded during an experiment in which a participant plays a game with ostensibly another participant (a confederate) via a video connection. However, in reality there is no live video connection and the original addressee plays the game with a pre-recorded video of the confederate (‘the speaker'). The illusion of a real connection, which typifies o-cam experiments, is facilitated by the use of specific techniques, see e.g., Goodacre and Zadro (2010). Contrary to experiments that involve physical presence of confederates, the o-cam paradigm allows for a tightly controlled environment where each participant is subjected to exactly the same speaker-stimulus, while having a highly ecologically valid setting. This particular o-cam experiment was executed by Brugel (2014) and aimed at eliciting listening behavior from the original addressees. Each original addressee played a Tangram game with the speaker, which consisted of 11 rounds.

**Figure 1:**
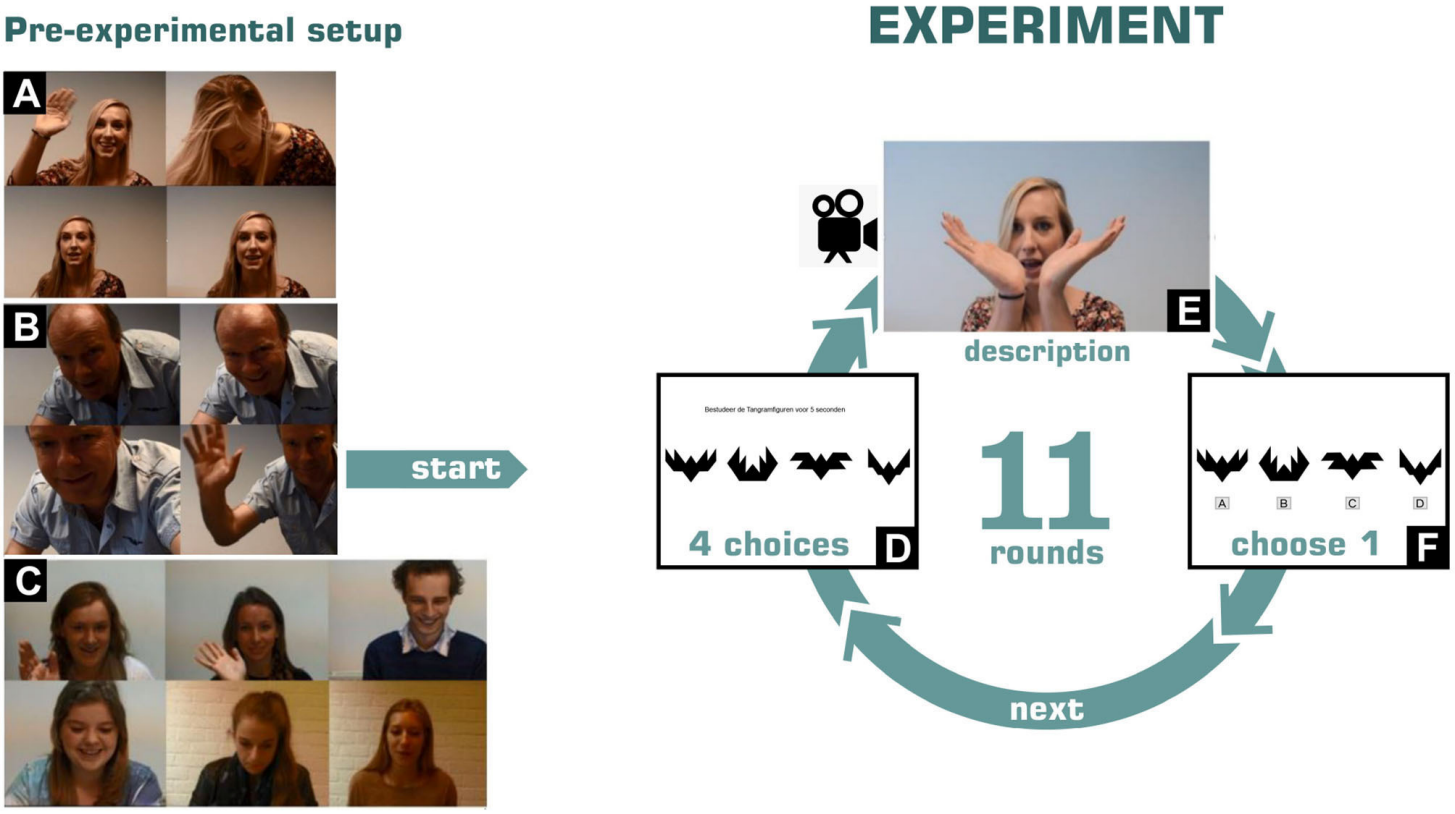
Visual impression of the o-cam experiment. First the participant is prepared **(A–C)**, after that 11 rounds are played: In each round the participant is shown 4 figures **(D)**, followed by a description of 1 of those figures **(E)**, and after which participant indicates which figure is described **(F)**.

In each round, the original addressee was first shown 4 different abstract pictures (tangram figures) for 5 s, subsequently, the speaker gave a description of one of those four tangram figures, and after that the original addressee had to choose which of the four shown tangram figures was described by the speaker. Each round contained 4 new tangram figures. See Figure 1 for an illustration of the Tangram game and experiment.

The experiment resulted in the video recordings of 14 original addressees, who each interacted with exactly the same speaker-stimulus. Each video was 8 min and 42 s long, and contained 6 min and 15 s of interaction. All videos were analyzed with Facereader (Noldus, 2019) to annotate the videos for a number of action units and head position. Head movements (nods) were then derived from the head pitch over time and quantified in terms of amplitude (maximum distance between y coordinates of the head position) and frequency (number of peaks and valleys of head position per timeframe). Sound was annotated by 1 coder in a binary fashion, 1 for presence of sound, 0 for being silent. The BOPs were identified by a panel of 10 judges. Each judge separately indicated the points in the speaker-stimulus that s/he thought were a BOP. With help of the parasocial consensus sampling method (Huang et al., 2010), all judgements were aggregated. All BOPs that were indicated by at least 3 judges were marked as genuine BOPs, which resulted in 53 different BOPs. See Blomsma et al. (submitted) for a detailed explanation of the data annotation process. The following two experiments make (indirect) use of the materials collected in that earlier dataset. Experiment 1 explores to what extent the perceived personality of the recorded human participants is determined by variation in their feedback behavior as compared to the personality perceived by appearance only. Experiment 2 tests to what extent findings from the first experiment generalizes to the perception of artificial avatars, whose feedback behavior was modeled based on the outcome of experiment 1.

## 3. Experiment 1: Perceived Personality of Real Humans

### 3.1. Method

#### 3.1.1. Participants

Eighty-two students from Tilburg University were recruited from the Tilburg University subject pool to participate in the first experiment in exchange for course credits. Seven students did not finish the experiment for unknown reasons and were discarded. The remaining 75 students did complete the experiment (11 male, 63 female and 1 other, Age: mean 21.31, SD = 3.17). The Research Ethics and Data Management Committee of the Tilburg School of Humanities and Digital Sciences approved the experiment under identification code REDC#2021/33. All participants provided their consent before they participated in the experiment.

#### 3.1.2. Stimuli

The stimuli consisted of 14 pictures, one of each original addressee, and 42 video clips of 8 s length. Pictures were included to get the baseline personality indication from participants (called *preconception score*), as personality impressions are likely to be based on mere appearance (Naumann et al., 2009). In order to get personality perception scores for all original addressees, while keeping duration of the experiment within reasonable limits, we choose to include the behavior of all original addressees while including only three specific BOPs.

**Figure 2:**
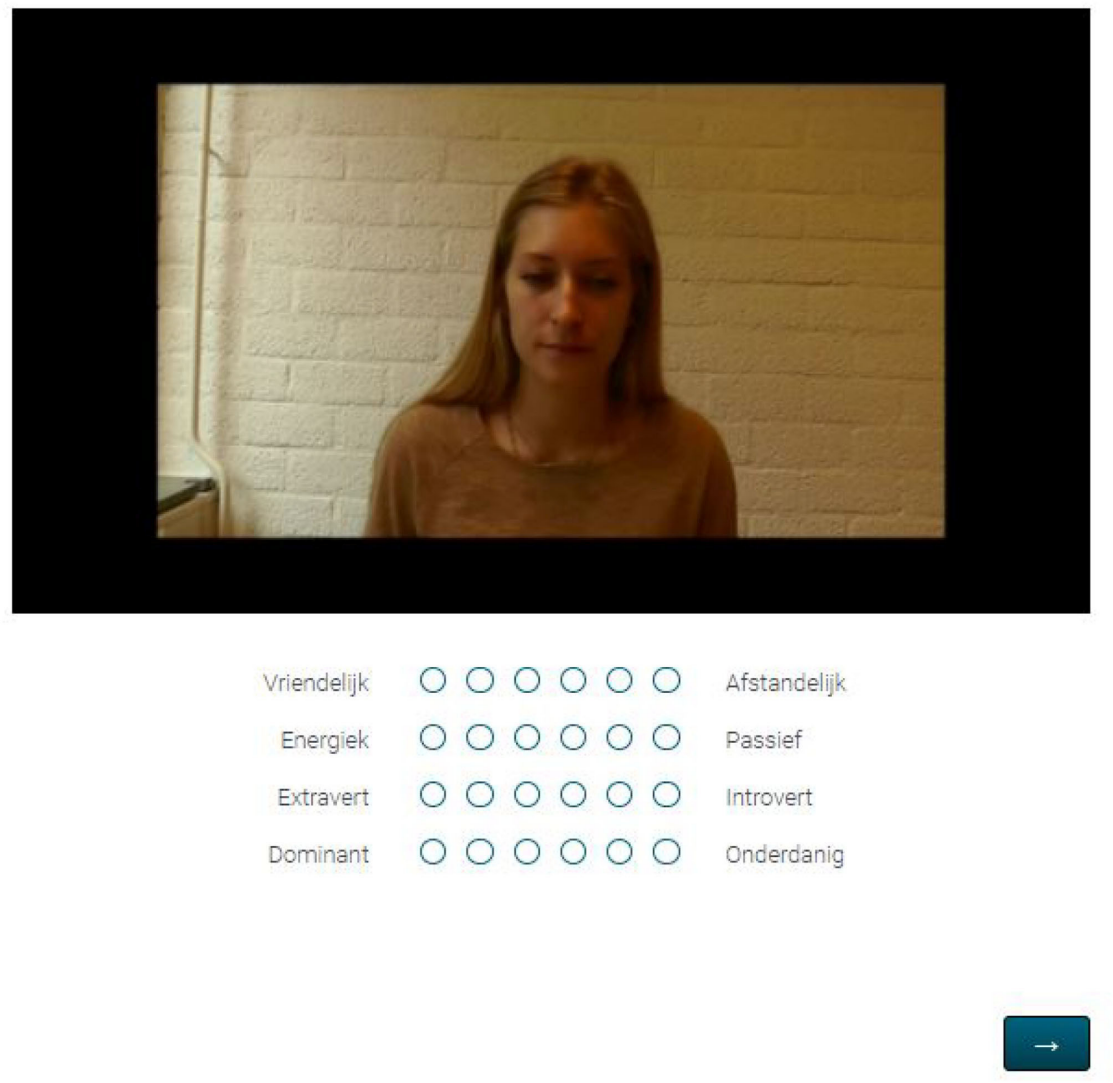
Impression of a neutral picture question as part of experiment 1.

The pictures were created by exporting the first neutral frame from each original addressee recording. Neutral here means that original addressee did not have any facial muscle contractions, in other words, all annotated AUs had a value of 0 for the exported frame. For one of the original addressees (1g) such neutral frame was not available in the dataset, as AU43 ('eyes closed') was annotated as contracted for many of the frames. The selected picture for this original addressee was the first neutral frame, with ignorance of AU43. See Figure 2 for a representative example of such neutral picture.

**Figure 3:**
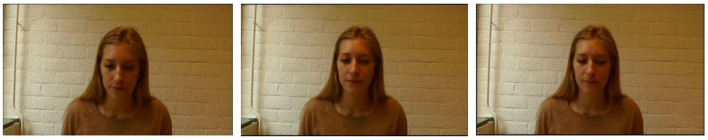
A few still images from an original addressee during BOP 49.

The video clips were extracted as follows: for each of the 14 original addressee recordings, three stimulus-videos of 8 s length were cut out, such that each video included a specific BOP. The stimulus-videos were cut such that the middle frame corresponded to the middle frame of the BOP. This resulted in 42 stimulus videos, each containing the behavior of one of the 14 original addressees for one of the three specific BOPs. See Figure 3 for an impression of a stimulus-video. The three selected BOPs were chosen from a pool of 53 BOPs. The decision for those three BOPs was guided by the desires that (1) each BOP would have a different characteristic and (2) that each original addressee would exhibit different behaviors among themselves during the selected BOPs. After manually trying out different combinations, the following BOPs were selected: 16, 47, and 49.

**BOP 16** takes place at the start of round 4, just after a short sentence of the speaker “These are more like birds.” and has a duration of 1,160 ms. The speaker has not shared much information at this point, and it is clear that she will need to share more information to enable the listener to identify the correct Tangram figure. Therefore, it may be inappropriate for the original addressee to provide a strong (vocalized) feedback signal as more information is coming and simply acknowledging to the speaker that one is listening seems to suffice. Interestingly, none of the original addressees vocalized during BOP 16. **BOP 47** occurs near the end of round 10, just after the speaker utters “The person looks to the left and the arms also point to the left.” and before the speakers says “I think you can figure it out by now.” BOP 47 is 920 ms long. At this feedback point it should be clear that the speaker shared all information that is needed to determine the correct Tangram figure, and thus a stronger feedback signal from the original addressee could be appropriate to signal that all information is understood. Six original addressees gave vocalized feedback signal at this BOP. **BOP 49** is approximately halfway round 11 (the last round) and has a length of 960 ms. Just after the speaker explained “You must have the one with the very lowest passage.” and before the speaker said “Thus, it's four buildings, all four with a sort of passage through in the middle …”. At this point in time the speaker explained the main hint that's needed for choosing the correct Tangram figure, however, the explanation is a bit ambiguous, hence the explanation that follows BOP 49.

**Figure 4:**
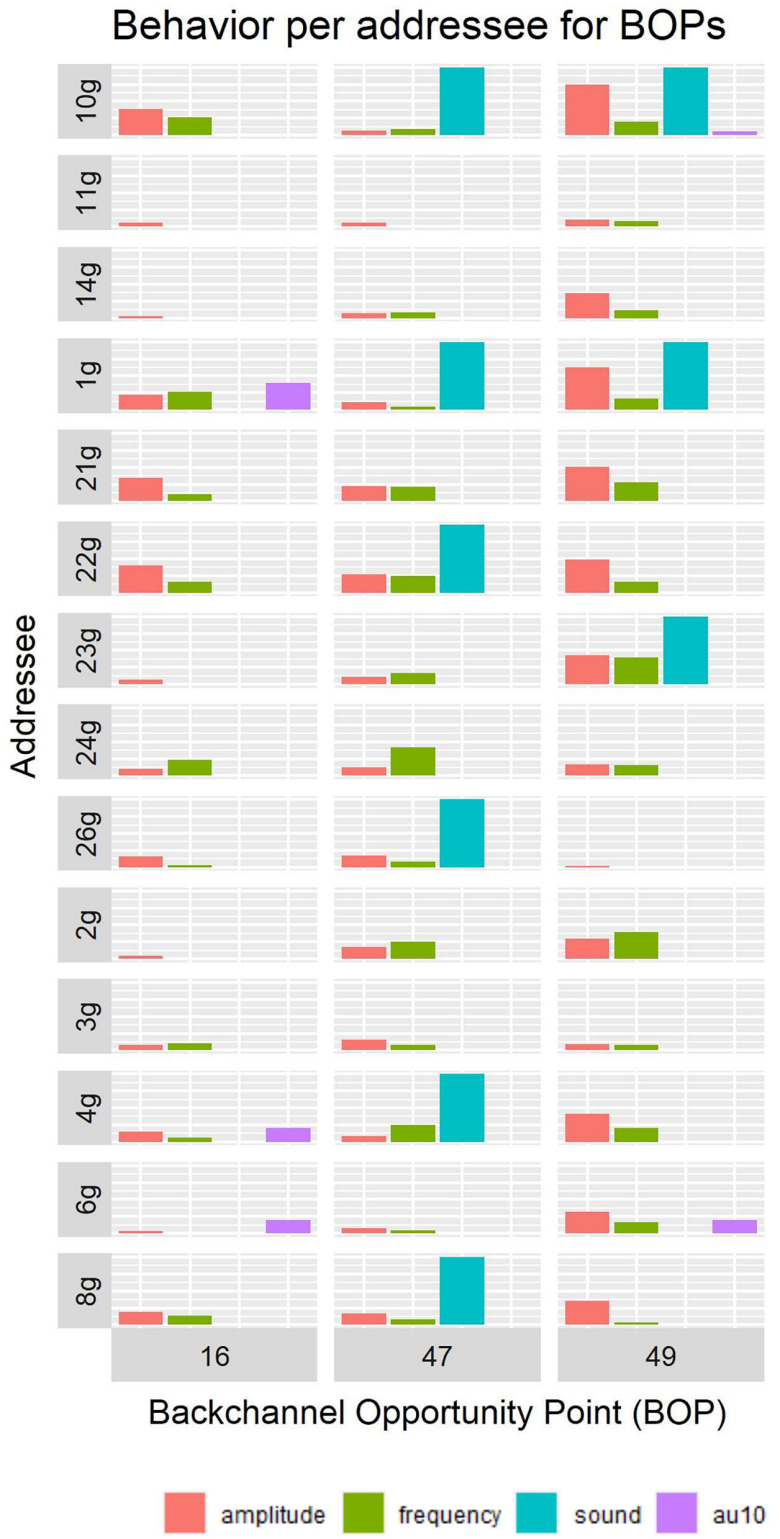
Quantification of the feedback behavior of the original addressees during the BOP 16, 47, and 49.

For an overview of the behavior of all original addressees during these three BOPs, see Figure 4.

#### 3.1.3. Procedure

Participants took part in an online experiment using the environment of Qualtrics (Qualtrics, 2021). Before the start of the experiment, participants read the instructions, signed the consent form and familiarized themselves with the task with one practice picture and one practice video. The practice stimuli were taken from the stimuli used in Vaitonyte et al. (2019) and were not in any way related to the stimuli used in the rest of the experiment. Successively, the stimuli (pictures and videos) were presented to the participants. First, the 14 pictures were presented in random order, followed by the presentation of the 42 video clips in random order as well. For each stimulus, participants were asked to rate the perceived personality of the original addressee in that stimulus for four dimensions on 6-point bipolar Likert scales: **Friendliness** (1:Friendly-6:Distant), **Activeness** (1:Active-6:Passive), **Extraversion** (1:Extravert-6:Introvert) and **Dominance** (1:Dominant-6:Submissive). See Figure 2 for an example of a presented stimulus.

The experiment concluded with two general questions: the participant was asked to fill in their age (open field) and indicate their gender (options: Male, Female, Other, Don't want to say). On average it took 42 min and 39 s to complete the experiment (SD = 151 min and 42 s).

#### 3.1.4. Statistical Analyses

Statistical analyses were conducted in R Studio (version 1.1.456; R Core Team, 2013). Linear mixed-effects models were used to fit each of the four personality dimensions with the lme4 package (Bates et al., 2014). The participant's response for each dimension served as the dependent variable. The main goal was to determine if behavior related to feedback contributed to the perception of personalities of original addressees or not. Therefore, we analyzed both the perceived personality of the original addressee in the static picture (called the preconception score), and the contribution of the audiovisual behavior during the BOP (sound, head movement (frequency and amplitude) and AU10). The fixed effects that entered the model were preconception score (6 levels: 1–6), sound (2 levels: sound, no-sound), frequency (number of nods per frame, value between 0 and 1), amplitude (maximum amplitude of nod per BOP in degrees, values between 0 and 28) and AU10 (mean contraction of AU10 during BOP, values between 0 and 5). Participants, BOPs and original addressees were treated as random effects, with random intercepts, in all models. Degrees of freedom and Satterthwaite approximation for *p*-values for all main effects were obtained from the lmerTest package (Kuznetsova et al., 2017). For every dimension we fitted two models, one with *preconception score* to see to what extent the static picture effects perception, and one without *preconception score* to see to what extent the more dynamic audiovisual features can explain the perceptual results on their own.

**Figure 5:**
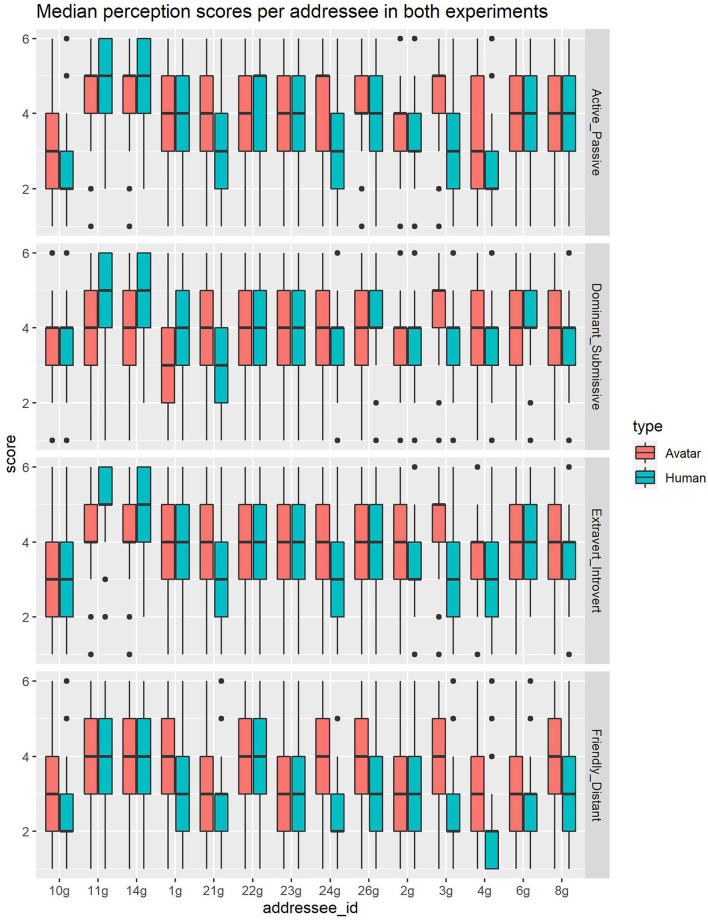
Overview of perception scores for all original addressees in experiment 1 and 2.

Table 1 contains the descriptive statistics of the perception scores per dimension, Figure 5 shows the perception scores per dimension and original addressee, and Table 2 contains the estimates for all fixed effects.

**Table 1:** Average scores per dimension for experiment 1 and 2.

	**Experiment 1**				**Experiment 2**	
	**Preconception**		**Video**		**Video**	
**Dimension:**	**Mean**	**SD**	**Mean**	**SD**	**Mean**	**SD**
Friendly-Distant	3.25	1.28	3.05	1.39	3.61	1.45
Active-Passive	3.63	1.27	3.61	1.42	3.99	1.37
Extroversion-Introversion	3.66	1.32	3.78	1.32	3.98	1.29
Dominant-Submissive	3.59	1.25	3.89	1.20	3.87	1.31

**Table 2:** Overview of fixed effects for all 12 models.

	**Human (all)**				**Human (without preconception score)**				**Avatar**			
	***b***	***SE***	***df***	***t***	***b***	***SE***	***df***	***t***	***b***	***SE***	***df***	***t***
**Friendly** **Distant**												
Intcpt	2.627	0.225	18.438	11.650^***^	3.341	0.230	16.676	14.501^***^	3.986	0.212	6.374	18.777^***^
Pre. s.	0.220	0.018	3128.955	11.971^***^	-	-	-	-	-	-	-	-
Amp.	–0.028	0.006	871.686	–4.657^***^	–0.028	0.006	816.973	–4.547^***^	–0.033	0.007	1654.109	–5.064^***^
Freq.	0.144	0.279	2797.100	0.515	0.145	0.285	2786.093	0.509	–0.417	0.308	2506.289	–1.354
Sound	–0.243	0.070	2554.217	–3.472^***^	–0.244	0.072	2519.544	–3.412^***^	–0.100	0.078	2677.430	–1.290
AU10	–0.492	0.145	2858.921	–3.383^***^	–0.493	0.148	2857.302	–3.322^***^	–0.206	0.080	2434.854	–2.578^*^
**Active** **Passive**												
Intcpt	3.176	0.198	23.348	16.058^***^	4.029	0.213	17.664	18.919^***^	4.389	0.174	8.275	25.295^***^
Pre. s.	0.235	0.018	3138.523	12.726^***^	-	-	-	-	-	-	-	-
Amp.	–0.027	0.006	115.228	–4.874^***^	–0.027	0.006	105.419	–4.762^***^	–0.037	0.006	1095.954	–6.031^***^
Freq.	–0.821	0.274	1589.229	–3.001^**^	–0.821	0.280	1528.568	–2.928^**^	0.042	0.286	2237.498	0.147
Sound	–0.311	0.068	988.773	–4.542^***^	–0.311	0.070	937.573	–4.448^***^	–0.404	0.072	2394.651	–5.593^***^
AU10	–0.207	0.143	1828.159	–1.452	–0.207	0.146	1776.930	–1.415	–0.120	0.074	2160.367	–1.608
**Extravert** **Introvert**												
Intcpt	3.057	0.164	25.761	18.680^***^	4.056	0.190	17.038	21.370^***^	4.315	0.136	7.991	31.687^***^
Pre. s.	0.273	0.017	3131.610	15.943^***^	-	-	-	-	-	-	-	-
Amp.	–0.020	0.005	56.902	–3.747^***^	–0.019	0.005	48.743	–3.608^***^	–0.030	0.006	530.140	–5.410^***^
Freq.	–0.391	0.259	1055.201	–1.510	–0.387	0.269	961.971	–1.437	0.228	0.264	1011.031	0.863
Sound	–0.264	0.065	568.042	–4.087^***^	–0.266	0.067	512.057	–3.961^***^	–0.495	0.067	1355.399	–7.381^***^
AU10	–0.040	0.135	1258.826	–0.295	–0.042	0.140	1167.998	–0.301	–0.115	0.069	929.692	–1.684
**Dominant** **Submissive**												
Intcpt	2.968	0.115	49.393	25.836^***^	4.020	0.138	18.585	29.218^***^	3.990	0.125	11.195	31.945^***^
Pre. s.	0.293	0.017	2975.232	17.188^***^	-	-	-	-	-	-	-	-
Amp.	–0.009	0.004	2586.582	–2.272^*^	–0.009	0.004	2988.443	–2.201^*^	–0.013	0.006	290.697	–2.273^*^
Freq.	–0.260	0.234	2267.288	–1.112	–0.241	0.247	2898.954	–0.977	0.508	0.272	693.490	1.870
Sound	–0.101	0.057	2233.669	–1.780	–0.103	0.060	2887.389	–1.706	–0.389	0.069	906.322	–5.630^***^
AU10	–0.111	0.122	2049.164	–0.905	–0.114	0.129	2825.401	–0.880	–0.004	0.070	638.318	–0.055

*The number of asterisks indicates p-level:*

*** *< 0.001,*

** *< 0.01,*

* *< 0.05*.

### 3.2. Results

#### 3.2.1. Friendliness

**Figure 6:**
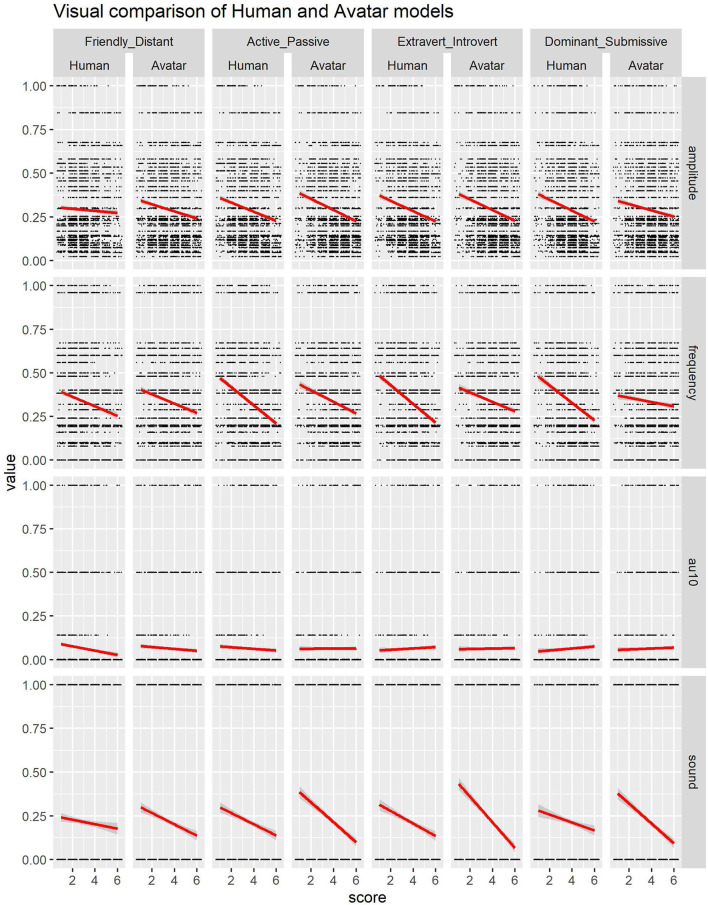
Visual indication of distributions for all variables available in Avatar model.

The average score given for the videos for the friendly-distant dimension was 3.05 (SD = 1.39), the videos of original addressee 4 g were perceived as most friendly (mean = 1.91, SD = 1.02), while the videos of original addressee 11 g was perceived as most distant (mean = 4.25, sd = 1.27). The *preconception score*, i.e., the score of the pictures of the original addressees, were rated on average with a 3.25 score (SD = 1.28). Similar to the results for the videos, the picture of original addressee 4 g was perceived as most friendly (mean = 2.27, sd = 0.90), while original addressee 21 g was perceived as most distant (mean = 4.23, SD = 1.10). The model with *preconception score* showed that *preconception score* had a significant effect on this dimension (*b* = 0.220, *SE* = 0.018, *df* = 3129.000, *t* = 11.971, *p* <0.001). However, also *amplitude* (*b* = –0.027, *SE* = 0.006, *df* = 871.700, *t* = –4.657, *p* <0.001), *sound* (*b* = –0.243, *SE* = 0.070, *df* = 2554.000, *t* = –3.472, *p* <0.001) and *au10* (*b* = –0.246, *SE* = 0.073, *df* = 2859.000, *t* = –3.383, *p* <0.001) had a significant effect on the perception of friendliness. The model without *preconception score* showed significant effects for the same variables: *amplitude* (*b* = –0.028, *SE* = 0.006, *df* = 816.900, *t* = –4.547, *p* <0.001), *sound* (*b* = –0.244, *SE* = 0.072, *df* = 2520.000, *t* = –3.412, *p* <0.001) and *au10* (*b* = –0.246, *SE* = 0.074, *df* = 2857.000, *t* = –3.322, *p* <0.001). From this we can conclude that, although appearance as tested with the *preconception score* played a significant role, that the amplitude, sound and AU10 during a BOP influence the perception of the friendly-distant dimension, such that a higher amplitude, usage of sound and more contraction of AU 10 correlates with a higher friendliness score. See also Figure 6 for a visual representation.

#### 3.2.2. Activeness

On average, participants rated the videos 3.61 (SD = 1.42) on the active-passive dimension. Like with the friendliness dimension, original addressee 4 g was perceived most active (mean = 2.35, SD = 1.51), while original addressee 11 g was rated as most passive (mean = 4.93, SD = 1.05). The mean *preconception score* was 3.63 (SD = 1.27), where the picture of original addressee 4 g was rated as most active (mean 2.41, score = 0.94) and original addressee 26 g as most passive (mean = 4.44, SD = 1.03). The model that included *preconception score* produced significant effects for *preconception score* (*b* = 0.235, *SE* = 0.018, *df* = 23.350, *t* = 12.726, *p* <0.001), thus appearance had a significant influence on the score. Next to that, *amplitude* (*b* = –0.027, *SE* = 0.006, *df* = 115.200, *t* = –4.874, *p* <0.001), *frequency* (*b* = –0.821, *SE* = 0.273, *df* = 1589.000, *t* = –3.001, *p* <0.001), *sound* (*b* = –0.311, *SE* = 0.068, *df* = 988.800, *t* = –4.542, *p* <0.001) had significant effects. When *preconception score* was ignored, the model also resulted in significant effects for *amplitude* (*b* = –0.027, *SE* = 0.006, *df* = 105.400, *t* = –4.762, *p* <0.001), *frequency* (*b* = –0.821, *SE* = 0.280, *df* = 1529.000, *t* = –2.928, *p* <0.001), *sound* (*b* = –0.312, *SE* = 0.070, *df* = 937.600, *t* = –4.448, *p* <0.001). So nodding behavior (amplitude and frequency) and sound both correlate with the perception of activeness. Frequent nodding, a higher amplitude and vocalizations during BOPs result in a higher activeness score.

#### 3.2.3. Extroversion

The videos were rated on average 3.78 (SD = 1.32) for the extroversion-introversion dimension. The videos of original addressee 4 g were rated as most extrovert (score = 2.35, SD = 1.15), while the videos of original addressee 14 g were rated as most introvert (mean = 4.85, SD = 1.03). The *preconception score* was 3.66 (SD = 1.32) on average, where original addressee 4 g was perceived as most extrovert (mean = 2.84, SD = 1.21) and 11 g as most introvert (mean = 5.11, SD = 0.90). The model extroversion-introversion score that included the *preconception score* produced significant results for *preconception score* (*b* = 0.273, *SE* = 0.017, *df* = 3132.000, *t* = 18.680, *p* <0.001), but also for the behavior related variables: *amplitude* (*b* = –0.020, *SE* = 0.005, *df* = 56.900, *t* = –3.747, *p* <0.001), *sound* (*b* = –0.264, *SE* = 0.065, *df* = 568.000, *t* = –4.087, *p* <0.001). The model for the extroversion-introversion score (intercept: 4.056, SE: 0.190) without preconception score produced also significant effects for *amplitude* (*b* = –0.019, *SE* = 0.005, *df* = 48.740, *t* = –3.608, *p* <0.001) and *sound* (*b* = –0.266, *SE* = 0.067, *df* = 512.100, *t* = –3.961, *p* <0.001). Thus, amplitude and sound influence, next to the appearance of the person, the extroversion - introversion score. A higher amplitude, and the presence of sound correlate with a higher score for extroversion.

#### 3.2.4. Dominance

The score for the videos was 3.89 (SD = 1.20) for the dominant-submissive dimension. Original addressee 21 g was perceived as most dominant (mean = 3.10, SD = 1.06), as based on the videos. Original addressee 11 g was perceived as most submissive (mean = 4.88, SD = 1.09). Preconception score was on average 3.59 (SD = 1.25), with original addressee 21 g being most dominant (mean = 2.37, SD = 1.11) and original addressee 11 g most submissive (mean = 4.79, SD = 0.87). The model with *preconception score* showed significant effects for *preconception score* (*b* = 0.932, *SE* = 0.017, *df* = 2975.000, *t* = 17.188, *p* <0.001) and *amplitude* (*b* = –0.009, *SE* = 0.004, *df* = 2587.000, *t* = –2.272, *p* <0.05). The model without *preconception score* showed also significant results for *amplitude* (*b* = –0.008, *SE* = 0.004, *df* = 2988.000, *t* = –2.201, *p* <0.05). While appearance has a significant correlation, amplitude also correlates with dominance: higher amplitude correlates with a higher perceived dominance.

### 3.3. Discussion

Our first experiment thus brought to light that the feedback behaviors significantly influenced the perceived personality of recorded participants, albeit that the relative importance of the variables we entered in our model varied as a function of the personality dimension we explored. Importantly, we showed that a person's personality is not merely based on the first impression we get from a still image, e.g., whether an individual shown in a picture at first sight looks friendly or dominant, but that this perception is modulated by more dynamic auditory of visual cues of that person. Our next experiment tests whether the findings based on analyses of real humans can be reproduced with avatar stimuli, in which various feedback behaviors are implemented.

## 4. Experiment 2: Perceived Personality of Avatars

### 4.1. Method

#### 4.1.1. Participants

Eighty-four students from Tilburg University were recruited from the Tilburg University subject pool to participate in the second experiment in exchange for course credits. Ten students did not complete the experiment for unknown reasons. Seventy-four students completed the experiment (20 male, 55 female, Age: mean 21.12, SD = 2.25). None of those participants had participated in experiment 1. The experiment was approved by the Research Ethics and Data Management Committee of the Tilburg School of Humanities and Digital Sciences under the same identification code as experiment 1 (REDC#2021/33). All participants gave their consent before participation.

#### 4.1.2. Stimuli

**Figure 7:**
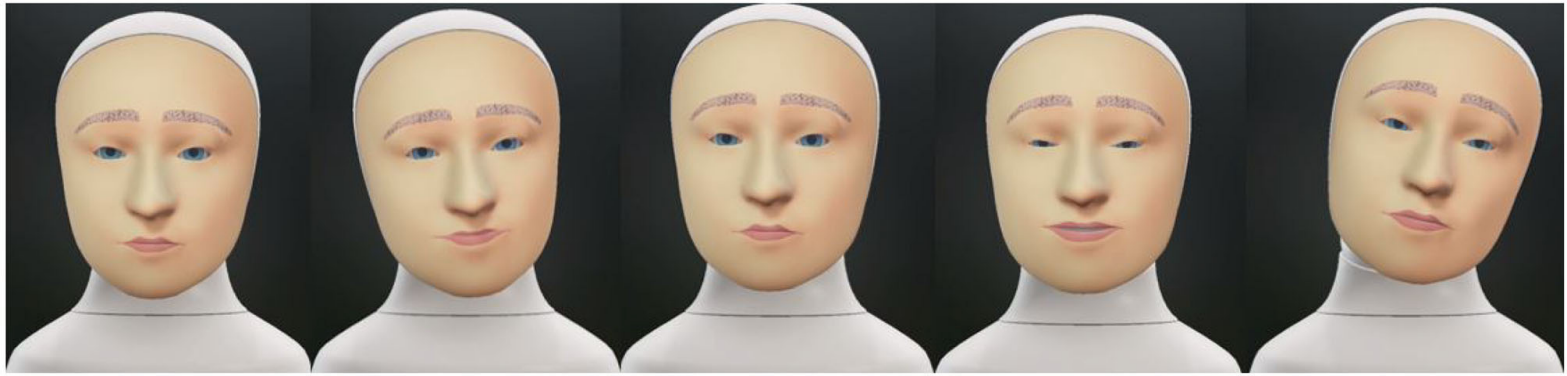
Visual impressions of the visual Furhat robot, as used during experiment 2.

The avatar experiment contained 42 videos, and contrary to the human experiment, did not include any still pictures. This was done because all videos in this experiment contained the same avatar, thus no preconception score was required. The content of the stimulus-videos of this experiment was exactly the same as those of the first experiment, except that the behavior of the original addressee in the original movie is now acted out by an avatar. The audio was copied from the original recordings. The avatar videos were created with the Furhat SDK (Al Moubayed et al., 2012), which provides a virtual simulation of the physical Furhat robot. First, the facial behavior of the original addressee was transferred onto the virtual Furhat robot. This was done by playing the original videos on a computer screen while having them analyzed on an IPhone with Live Link Face app (version 1.1.1). Live Link Face analyzes 62 different properties of facial behavior (including head movement) on a 60 frames per second basis. The output of Live Link Face was then converted with the Furhat Gesture Capture app (version 4.3.6) and played out on the virtual Furhat using Furhat SDK 2.0.0^1^. The Furhat SDK offered a collection of 10 different avatar-faces, so called textures. All sequences were played out on the default texture. Having the same face for all sequences had the advantage that texture-specific effects did not have to be taken into account. The default texture was, in our opinion, the most gender-neutral option from the collection, such that it would work for sequences originating from both genders. In addition, compared to other textures, the default texture has a rather cartoonish appearance which would minimize the chance of a uncanny valley related experience among the participants. Furhat was recorded with the OBS screencapture tool (version 27.0.1)^2^. The 14 recordings were then synchronized and merged with the sound of the original video with ShotCut (version 21.01.29)^3^. From here, we used the same method as in the human experiment. We cut out three videos per avatar video, each containing the original addressees behavior during exactly one BOP. Figure 7 shows a few still images from Furhat as appearing in the stimuli.

#### 4.1.3. Procedure

Participants again took part in the online experiment using the online environment of Qualtrics (Qualtrics, 2021). Before the start of the experiment, participants read the instructions, signed the consent form and familiarized themselves with the task with two practice videos. The practice video clips of Furhat were created in the same fashion as the stimuli for the experiment, but using a different BOP (BOP 21). Participants were asked to watch the video clips and indicate how they judged the personality of the original addressee in the same way as described for Experiment 1. The 42 video clips were shown in random order. On average it took 18 min and 46 s to complete the experiment (SD = 19 min and 34 s).

#### 4.1.4. Statistical Analyses

The results of experiment 2 are analyzed in the same way as experiment 1. However, as experiment 2 did not include stimuli to obtain a *preconception score*, the results contain only one model.

### 4.2. Results

#### 4.2.1. Friendliness

On average, Furhat received a score of 3.61 (SD=1.45) for the friendly-distant score. The transferred behavior of original addressee 10 g was perceived as most friendly (mean = 2.75, SD = 1.33), and 14 g as most distant (mean = 4.24, SD = 1.28). The model for the friendly-distant score (intercept: 3.986, SE: 0.212) produced significant effects for *amplitude* (*b* = –0.033, *SE* = 0.007, *df* = 1654.000, *t* = –5.064, *p* <0.001) and *au10* (*b* = –0.206, *SE* = 0.080, *df* = 2435.000, *t* = –2.578, *p* <0.05).

#### 4.2.2. Activeness

The videos were rated, on average, with a score of 3.99 (SD = 1.37) on the active-passive scale. The behavior of original addressee 10 g was perceived as most active (mean = 3.10, SD = 1.36), while that of original addressee 14 g was perceived as most passive (mean = 4.57, SD = 1.15). The model for the active-passive score (intercept: 4.389, SE: 0.174) produced significant effects for *amplitude* (*b* = –0.037, *SE* = 0.006, *df* = 1096.000, *t* = –6.031, *p* <0.001) and *sound* (*b* = –0.404, *SE* = 0.072, *df* = 2395.000, *t* = –5.593, *p* <0.001).

#### 4.2.3. Extroversion

The mean score for extroversion-introversion was 3.98 (SD = 1.29). The most extraverted behavior was that of original addressee 10 g (mean = 3.30, SD = 1.32) and the behavior of original addressee 3 g was perceived as most introverted (mean = 4.48, SD = 1.11). The model for the extroversion-introversion score (intercept: 4.315, SE: 0.136) produced significant effects for *amplitude* (*b* = –0.030, *SE* = 0.006, *df* = 530.100, *t* = –5.410, *p* <0.001) and *sound* (*b* = –0.495, *SE* = 0.067, *df* = 1355.000, *t* = –7.381, *p* <0.001).

#### 4.2.4. Dominance

The mean perception for dominant-submissive was 3.87 (SD = 1.31). Original addressee 1 g was perceived as most submissive (mean = 3.46, SD = 1.44) and that of original addressee 3 g as most dominant (mean = 4.47, SD = 1.16). The model for the extroversion-introversion score (intercept: 3.990, SE: 0.124) produced significant effects for *amplitude* (*b* = –0.013, *SE* = 0.006, *df* = 290.697, *t* = –2.273, *p* <0.05), *sound* (*b* = –0.389, *SE* = 0.069, *df* = 906.322, *t* = –5.630, *p* <0.001).

### 4.3. Discussion

On a general level, our second experiment with judgments of avatars replicated the results of our first experiment in which human beings were being rated, in the sense that variable feedback behaviors again led to differences in perceived personality of the avatars. However, we also noticed that the results of both experiments were slightly at variance regarding the significance and strength of specific auditory and visual cues. Although we have not included a statistical comparison between the models in this paper, we will discuss the differences between the models in more detail in the general discussion section.

## 5. General Discussion and Conclusion

We have reported about two perception experiments, both consisting of 42 8-s video clips that showed a human or artificial listening original addressee. Participants were asked to rate the perceived personality of that original addressee in terms of different dimensions. The first experiment also presented participants with still images of the original addressees in the clips, who were likewise rated regarding the different personality traits. In the first experiment, the video clips contained 14 different original addressees during 3 different backchannel opportunity points (the moments in conversation that allow for feedback). In the second experiment, the same stimuli were shown, except that they were re-enacted by a virtual Furhat robot. The results of the first experiment showed that backchannel behavior influences personality perception, which modulated the first impressions that people obtained from the still pictures. The results of the second experiment show that comparable effects could be achieved when such behavior is re-enacted by a conversational AI system. In the following, we first detail more specific resemblances and differences between the outcomes of the two experiments, and then discuss the outcomes in a broader perspective.

An overview of the significant results from the various models can be found in Table 2. While the results are quite analogous for ratings of human and artificial stimuli, we also observe some variability. Regarding the Friendly—Distant dimension, we see that the model of for the human condition produced significant results for amplitude, sound and AU10, while the avatar model only did so for amplitude and AU10. Moreover, the estimate for AU10 for the human condition (–0.493) is more than double the estimate for the avatar condition (–0.206). For the Active—Passive dimension, the human model produced significant results for frequency, but the avatar model did not generate any significant results. For the Extroversion - Introversion dimension, both models produced significant effects for the variables amplitude and sound, even if sound had a higher estimate for the avatar condition (–0.495) than for the human condition (–0.266). And finally, when looking at the Dominant—Submissive dimension, we see that the human condition only had a significant effect for amplitude, while the avatar model also had a significant effect for sound as well (next to amplitude).

So while the results appear to be quite consistent over the two experiments, it may be worthwhile to reflect somewhat on the differences between conditions, especially regarding the variable effect of sound. First, it is important to note that we focused on the effect of four sets of features in both human and artificial stimuli on personality perception, namely sound, amplitude, frequency and AU10. But while only these features were varying in the avatar data, the human data also contained additional variation that we had not investigated further (e.g., other facial expressions, hand gestures and body posture) that nonetheless could have affected the perception results, if only because they made the human data more natural. In that sense, the conditions are not entirely comparable, as judgments of human data may be closer to what people do in their daily life than judgments of artificial creatures.

Also, note that the audio variable is different from the visual features in that this one was identical in both conditions, whereas the visual features were modeled via the avatar settings, and therefore only a computational approximation of the human data. Yet, despite the similarity regarding the audio feature, it is interesting to observe that this variable does not always have similar effects in the human and avatar data on personality judgments. For instance, the audio data increase the perception of friendliness when human original addressees are judged, but not when the avatar data are scored. Maybe this could be due to the fact that participants, when rating this dimension of friendliness in avatar stimuli, are unsure about their judgments. Indeed, of the nine avatar videos that contained vocalizations, the friendliness perception scores of three of those videos are highly variant. Maybe this is due to the fact that judges have some difficulty to relate the natural voice with an artificial visual appearance of the avatar, so that they have problems taking the audio variable into account for this variable. Other factors may include the effects related to the mismatch between the human voice and the human-looking (but rather cartoonish) avatar, as non-human systems endowed with real human voice may lead to expectations mismatch (Moore, 2017). Moreover, it may have been somewhat confusing for participants to note that, although the visual appearance of the avatar was the same in all stimuli, the choice of voices changed.

Conversely, we observe a significant effect of the audio variable on the judgments of dominance with avatars, while this effect is absent in the judgments of human data. According to the literature, the dominance-submission is, in general, perceived through multiple channels: Facial expressions related to anger and aggression are perceived as highly dominant, while fearful expressions are related to submission (Hess et al., 2000), direct eye contact and upward head tilt express dominance, contrary to downward head tilt and averted gaze which are perceived as submissive (Mignault and Chaudhuri, 2003). Voice frequency is related to dominance as well, as men rate male voices with a lower frequency as more dominant (Puts et al., 2006). In that sense, the avatar data may have represented a relatively poor approximation of this dimension, as many variables mentioned above were not included in the stimuli, so that participants may have relied to a larger extent on the audio cue, compared with their judgments of the human data. Also, it is important to note that voice dominance is gender specific, as low voice has been shown to lead to perception of male voices only, whereas the Furhat character seems to look rather gender-neutral, which could have influenced perception as well. In a future study we could also include facial AUs related to expressions of fear and anger to see if those influenced the perception.

How can the insight that personality is perceived through backchannel behavior be integrated into the behavior of an ECA? In case the backchannel behavior of the ECA is modeled based on human data, we recommend selecting the humans based on the desired personality of the ECA, rather than modeling the behavior based data originating from humans with random personalities. So e.g., if the desired personality of an ECA is introverted, utilize the data of people that are perceived as introverted. Additionally, in case of adjusting an existing backchannel generation algorithm, we would focus on increasing or decreasing the amplitude. As amplitude showed a significant effect for all four personality dimensions, we expect that magnifying the head movements of the avatar would lead to a more extravert, friendly, active and dominant perceived ECA, while reducing the head movements would lead to the opposite perception. The exact magnitudes to increase or decrease the amplitude is part of future research.

In conclusion, personality perception is indeed influenced by the behavior a person exhibits during back channel opportunity moments. Especially the utilized amplitude for head nodding behavior correlates with multiple personality dimensions. These results suggest that it could be useful for conversational AI, and ECA developers in particular, to start implementing feedback behavior generation algorithms that take into account the reported variables (amplitude, frequency, sound and AU10) to strengthen the personality perception of their avatar in order to create more natural interactions and induce a stronger social presence with its interlocutors.

## Data Availability Statement

The raw data supporting the conclusions of this article will be made available by the authors. The videos, however, are not readily available because of privacy reasons of the filmed participants. Requests to access the dataset should be directed to the first author.

## Ethics Statement

The studies involving human participants were reviewed and approved by the Research Ethics and Data Management Committee of the Tilburg School of Humanities and Digital Sciences. The patients/participants provided their written informed consent to participate in this study. Written informed consent was obtained from the individual(s) for the publication of any potentially identifiable images or data included in this article.

## Author Contributions

PB conducted the experiments, performed the statistical analysis, and wrote the first draft of the manuscript. MS wrote sections of the manuscript. All authors contributed to conception and design of the study, revision, read, and approved the submitted version.

## Conflict of Interest

The authors declare that the research was conducted in the absence of any commercial or financial relationships that could be construed as a potential conflict of interest.

## Publisher's Note

All claims expressed in this article are solely those of the authors and do not necessarily represent those of their affiliated organizations, or those of the publisher, the editors and the reviewers. Any product that may be evaluated in this article, or claim that may be made by its manufacturer, is not guaranteed or endorsed by the publisher.

## Footnotes

^1^ https://www.furhat.io

^2^ https://www.obsproject.com

^3^ https://www.shotcut.org
